# Metabolic Comorbidities and Risk of Development and Severity of Drug-Induced Liver Injury

**DOI:** 10.1155/2019/8764093

**Published:** 2019-08-18

**Authors:** Xu Li, Pujun Gao, Junqi Niu

**Affiliations:** Department of Hepatology, The First Hospital of Jilin University, Jilin University, No. 71 Xinmin Street, Changchun 130021, China

## Abstract

The incidence and rates of diagnosis of drug-induced liver injury (DILI) have been increasing in recent years as findings from basic research and the examination of clinical databases reveal information about the clinical course, etiology, and prognosis of this complex disease. The prevalence of metabolic comorbidities (e.g., diabetes mellitus, fatty liver, obesity, and metabolic syndrome (MetS)) has been increasing during the same period. The results of preclinical and clinical research studies indicate that characteristics of metabolic comorbidities are also factors that affect DILI phenotype and progression. The objective of this review is to present the evidence for DILI and hepatotoxicity mechanisms, incidence, and outcomes in patients with MetS and nonalcoholic fatty liver disease. Moreover, we also summarize the relationships between drugs used to treat metabolic comorbidities and DILI.

## 1. Introduction

Liver damage may be caused by drugs or their metabolites, or both, during drug therapy [1]. This drug-induced liver injury (DILI) is a serious adverse drug reaction that may result in acute liver failure [2, 3]. Liver injury can be caused by >1100 currently available drugs [4, 5]. Due to the increase in available drugs and the irrational use of drugs, the numbers of DILI cases increased each year as a result of increases in drug availability and nonscience-based prescription of drugs. The World Health Organization found that DILI is the fifth most common cause of liver disease-associated death [6].

One still-controversial classification system subdivides DILI as ‘intrinsic' or ‘idiosyncratic' [7, 8]. Intrinsic DILI is dose-dependent and the disease course is, therefore, predictable. Some dose-dependency is associated with idiosyncratic DILI, but this type is mostly not dose-dependent and the disease course is unpredictable. Idiosyncratic DILI occurs in a small proportion of susceptible individuals experiencing idiosyncratic DILI with a prolonged latency. Patients affected by intrinsic DILI experience the drug-induced direct hepatotoxicity during a short period of a few days.

Risk factors of DILI (e.g., drug characteristics, the host's metabolic and immunological factors) are complex and interrelated. Females [9], the elderly [10], patients with underlying chronic liver disease [11], obesity, and HIV [12] are at greater risk for DILI. Drug characteristics (e.g., medication dose, extent of hepatic metabolism, and drug lipophilicity) also contribute to the DILI risk. Characteristics of the patient (e.g., viral infection, alcohol intake, and preexisting disease) are also important.

Limited evidence suggests that a higher body mass index (BMI) and metabolic comorbidities (metabolic syndrome (MetS), type 2 diabetes mellitus (T2DM), obesity, and nonalcoholic fatty liver disease (NAFLD)) contribute to DILI clinical presentation and outcome [13, 14]. Hepatic fat accumulation may reduce susceptibility to tissue injury if the liver is exposed to harmful stimuli (e.g., ischemia/reperfusion, bacterial endotoxins)[15]. However, there are few results of clinical studies that investigate the effects of metabolic comorbidities on DILI risk [16]. The aim of this review is to examine the evidence for DILI and hepatotoxicity mechanisms, incidence, and outcomes in patients with MetS and nonalcoholic fatty liver disease. Moreover, we also summarize the relationships between drugs used to treat MetS, diabetes and hyperlipidemia and DILI.

## 2. Biological Mechanisms Linking Metabolic Factors and DILI

Two key mechanisms are associated with DILI onset. First, whether exposure to a drug or metabolite results in reaching the threshold for liver injury is determined by the dose and hepatic processing metabolism. Second, the adaptive immune response (i.e., “alarm-signaling” by damage-associated molecular pattern molecules) is also important [17]. The way that defense mechanisms interact with the toxic drug exposure determines whether cell damage occurs. The adaptive and innate immune responses that react to cell damage have significant roles in the subsequent tissue inflammation and injury. The extent of local tissue inflammation and injury interacts with the tissue repair responses to affect the overall tissue damage and clinical outcomes. During the initial stages of cellular damage, the characteristics of the administered drugs and the extent of drug exposure have primary roles. After induction of cellular repair mechanisms, the host's factors drive the ‘host responses' to toxic insults.

Systematic investigations of the metabolic factors that contribute to an individual's susceptibility to, and the clinical phenotypes of, DILI have not been performed. The objective of this section of the review is to provide a cross-disciplinary discussion of host factors that affect the key mechanistic components of DILI; the four categories are drug processing, toxicological responses, inflammation and immune responses, and imbalances between tissue damage and induction of repair processes (Table 1).

### 2.1. Metabolic Factors That Affect Drug Processing

Lifestyle, disease conditions, and comedications also modify individual drug processing capability. The cytochrome P450s (CYP450s) 2E1 and 4A can be induced by high fat diets and alcohol. In humans, an increased risk of acetaminophen-induced hepatic injury is associated with CYP2E1 because this CYP induces increased generation of the reactive metabolite N-acetyl-p-benzoquinone imine (NAPQI) [18]. Meanwhile, increased activity of CYPs (CYP2C9, CYP1A2, CYP2E1, and CYP2D6) has been found in individuals with obesity; increased CYP activity could augment toxic metabolite generation [19, 20]. The greater hepatoxicity of halothane and acetaminophen in patients with obesity and NAFLD could be explained by greater CYP2E1 activity. This CYP transforms these drugs into the highly reactive metabolites trichloroacetyl chloride in individuals with obesity and into NAPQI in individuals with NAFLD [19, 21]. Decreased drug clearance and the subsequent slower elimination of drugs and higher drug levels in plasma are associated with high body fat [22].

### 2.2. Toxicological Responses Modified by Metabolic Factors

The diverse mechanisms via which drugs initiate cell damage include formation of reactive metabolites and subsequent covalent binding to cellular proteins, endoplasmic reticulum stress, oxidative stress, DNA damage, mitochondrial injury, epigenetic modification, and inhibition of bile acid excretion. Metabolic factors modify toxicological responses through processing mentioned above.

Drug-associated induction of cellular oxidative stress can represent a severe toxicological insult. Increases in the host's preexisting cellular oxidants and in the substrates used for oxidative reactions (e.g., steatosis, lipid peroxidation) and decreases in antioxidants can modify the severity of drug-induced oxidative insult. Cellular oxidative stress is also affected by other host factors: (1) obesity, insulin resistance, and NAFLD increase cellular oxidants, (2) fatty liver increases lipid peroxidation, and (3) obesity, insulin resistance, and NAFLD are related to cellular antioxidation (e.g., SOD2, GPx1[23]) depletion of antioxidants.

In patients with obesity, diabetes, insulin resistance, and nonalcoholic steatohepatitis, the metabolic environment characterized by reduced ATP synthesis and higher reactive oxygen species (ROS) levels can increase cytotoxicity [24–26]. When significant amounts of ROS are produced, they are usually appropriately detoxified when the mitochondrial biology is normal [27]. However, host factors such as alcohol consumption and overnutrition can affect the mitochondrial aging that is partly due to accumulated oxidative mitochondrial DNA damage [13, 28]. During NAFLD, higher ROS levels can result from reduced antioxidant defenses (e.g., glutathione peroxidases, glutathione, superoxide dismutases) and ROS overproduction by cellular components (e.g., respiratory chain, peroxisomes, CYP2E1)[25, 29].

Inhibition of bile acid transporter could lead to intrahepatocellular bile acid accumulation [30]. Hepatic transporters are influenced by genetic variations, comedications, bacterial endotoxins, and the farconoid xenosensing receptor (FXR), which functions as a bile acid sensor and acts as a key regulator of metabolic processes [31]. Some data have showed that decreased expression of hepatic FXR is associated with an increased expression of SREBP-1C, LXR, and hepatic triglyceride synthesis; furthermore, increased SREBP-1C is associated with the degree of hepatic steatosis in the NAFLD patients [32].

### 2.3. Metabolic Factors That Modulate Immune Response and Inflammation and Modify Cell Death, Tissue Injury, and Repair

The innate and adaptive immune responses have important roles in determining the degree of ‘injury' and inducing inflammation. Host factors are known to modulate the inflammation and immune responses which affect DILI susceptibility in turn.

Inflammation and immune responses are affected by sex hormones and gender. Using an immune-mediated DILI model, researchers found gender bias in inflammation and immune response. Compared with males, females had more severe hepatitis, more antibody production, and higher levels of proinflammatory hepatic cytokines [33]. An increased influx of lipopolysaccharide [34, 35], altered microbiomes [36], chronic inflammatory diseases and viral infections, obesity [37], progesterone [38], and depletion of the bile acid pool [39] interact synergistically to enhance hepatotoxic drug-associated liver damage. During nonalcoholic steatohepatitis, high tumor necrosis factor production occurs in the proinflammatory environment. Tumor necrosis factor can sensitize the liver to drug-induced acute cytotoxicity [35, 40].

These experimental results are suggestive, but the available study results for human populations provide little support for an overall increased risk of DILI in patients with diabetes, obesity, or NAFLD [14]. More studies are needed to understand the mechanisms that increase the toxicity of some drugs to the livers of patients with obesity. Meanwhile, the balance between tissue injury and repair also needs to be considered with impaired tissue repair worsening the condition leading to poor clinical outcome. Potential beneficial impacts of lipid-lowering drugs (i.e., statins, fibrates) are associated with improved clinical outcomes in patients diagnosed with dyslipidemia and collagen diseases among DILI cases [41].

Epigenetic modifications of host chromatin may impair regeneration following injury and potentially alter individual susceptibility to hepatotoxicity [42, 43]. Deficiencies of nutrition can cause epigenetic modifications. Deficiencies of choline, vitamin B12, and folic acid deficiency influence cellular metabolism, hepatocyte differentiation, and severe liver damage [44–46].

## 3. Epidemiological Studies Linking Metabolic Comorbidities and DILI Incidence and Severity

In obese individuals with NAFLD, drugs such as halothane that may still be used in some countries and as a generic product in the USA in children, isoflurane, acetaminophen, ticlopidine, losartan, and omeprazole can increase the risk of acute DILI [47–49]. Treatment with methotrexate (MTX) in patients with MetS increases the risk of MTX hepatotoxicity. Obesity and diabetes are identified as risk factors for MTX-induced liver injury by American Association of Dermatology and American College of Rheumatology guidelines [50]. With regard to DILI severity in patients with MetS or NAFLD, a recent DILI initiative study found that that preexisting liver disease is a risk factor for a more severe form of DILI at presentation and higher mortality [51].

However, two prospective, population-based studies did not suggest that the metabolic variables BMI, T2DM, or NAFLD were predisposing factors for DILI [9, 52]. In one of the populations (in France) these variables might not have been included in the study or were not available for study. In the other population with DILI (in Iceland), the values for prevalence of diabetes and obesity were low (5% and 10%, respectively)[9]. NAFLD or its shared metabolic risk factors are not usually mentioned by large prospective registries of DILI as determinants of increased odds of hepatotoxicity [13, 22, 53, 54]. The results of one clinical study suggested that the frequency of amiodarone-induced hepatotoxicity was not greater in patients with metabolic syndrome [55]. A series of 308 consecutive cases of acute liver failure admitted to referral centers was examined by the Acute Liver Failure Study group, but results on metabolic risk factors and clinical outcome were not included in the study findings [56].

Some of these studies were performed in populations with low values for the prevalence of T2DM (i.e., Iceland) and obesity. Also, MetS and NAFLD are usually not included as relevant variables in multicenter registry databases. The numbers of adverse events for individual drugs is usually small, so it is difficult to make valid conclusions. Therefore, the values for the strength of association of metabolic factors as risk factors for DILI might have been underestimated in these studies.

### 3.1. Obesity and NAFLD

A sedentary lifestyle and overconsumption of calories can at least partly explain the epidemics of obesity occurring in many countries. Because obesity increases the risk of many diseases (e.g., coronary heart disease, type 2 diabetes, NAFLD, and some cancers) the high prevalence of obesity is a major public health problem. Obesity is a risk factor for DILI, at least for some drugs [19, 57] (the volatile halogenated anesthetics halothane and isoflurane, acetaminophen, omeprazole, losartan, and ticlopidine,) [47, 49, 57, 58]. More severe hepatotoxicity occurs in obese rodents given isoflurane [19, 47, 59–62], tetracycline [63], phenobarbital [64], or haloperidol [65]. However, a poor acute liver failure outcome was not predicted by BMI in subjects with drug-induced acute liver failure who were on average overweight, but not obese [66]. Whether drug-induced acute liver failure was predicted by a high BMI (i.e., obese) was not examined in this study.

The findings of Tarantino et al. [48] suggested that compared with patients with hepatitis C virus infection, patients with NAFLD have a higher risk of DILI. Their study methodology was problematic, and their study population included only 6 cases of DILI among 74 patients with NAFLD and 1 case of DILI among 174 patients with hepatitis C virus infection. A study performed in Italy found that compared with patients with hepatitis C virus-associated chronic hepatitis, patients with NAFLD have a higher risk of DILI [67]. An odds ratio of 3.95 was found for drug-related hepatotoxicity in middle-aged patients with obesity and NAFLD, but only a small number of cases was included in this study. The results of clinical and experimental studies strongly suggest that acute acetaminophen hepatotoxicity is more severe and frequent in populations of patients with obesity and NAFLD [21, 49, 68, 69]. One prospective study found that middle-aged patients with obesity and preexisting NAFLD had a nearly fourfold increased risk of DILI [48]. There is greater appreciation of the potential increased risk of DILI in patients with NAFLD who are treated with drugs such as fosinopril, piperacillin/tazobactam, omeprazole, ticlopidine, or telithromycin [48]. A study of patients with preexisting liver disease (mainly hepatitis C virus infection and NAFLD)[51] found that the clinical severity of DILI tended to be higher and the lethality was significantly higher in individuals with these preexisting liver diseases [70–72].

The increased susceptibility to DILI in individuals with obesity might be associated with other mechanisms associated with drug availability or metabolism or inflammatory changes in adipose tissue that indirectly affect the liver, rather than to steatosis per se [73]. In patients with obesity and NAFLD, DILI could present one of two different clinical entities. In an obesity and related metabolic disease context, some drugs are more likely to cause worsening of preexisting NAFLD and others are more likely to induce an acute hepatitis.

Some drugs stimulate lipogenesis in a steatotic liver. Drug-induced worsening of NAFLD can also occur secondary to an increase in insulin resistance. This key mechanism results in hepatic lipid deposition. An increase in insulin resistance stimulates hepatic lipogenesis secondary to hyperinsulinemia, adipose triacylglycerol hydrolysis, and delivery of free fatty acids to the liver. Synthetic corticosteroids, NRTIs, antipsychotic drugs (e.g., olanzapine, clozapine), thiazide diuretics (e.g., hydrochlorothiazide), and protease inhibitors initiate or increase insulin resistance [57, 74–76].

Reduced mitochondrial-associated glutathione (GSH) levels [25] can negatively affect CYP-generated reactive metabolite removal. This mechanism can also account for the higher drug-induced acute hepatitis risk in patients with obesity. Obesity is associated with reduced activity of some CYPs (e.g., CYP3A4)[20]. Therefore, not all drugs that can be converted to toxic metabolites present an increased acute hepatitis risk. The increased glucuronosyltransferase activity that is more common in some patients with obesity [20, 21] can increase the rates of detoxification of some compounds. Underdosing might also occur with drugs whose dosages do not account for a higher body mass index.

The susceptibility of acute liver injury might not be increased by NAFLD for all hepatotoxic drugs. NAFLD-associated changes in xenobiotic metabolizing enzyme activity might not be associated with higher toxic compound (i.e., the parent drugs or the CYP-generated reactive metabolites, or both) levels [15].

### 3.2. Dyslipidemia and Diabetes Mellitus

Few studies of the links between dyslipidemia and DILI have been performed. Results of an analysis of the Spanish DILI registry [77] indicate that patients with baseline dyslipidemia have a relatively higher risk for chronic liver changes [odds ratio = 4.5, p = 0.04]. However, Robles-Diaz found that dyslipidemia is less frequent in patients with acute liver failure or orthotopic liver transplantation compared with patients with favorable outcomes. The apparent protective effect of dyslipidemia might be associated with the more frequent statin use by those with dyslipidemia (8.7% versus 3.2%, respectively)[78].

Some study results suggest that DM affects DILI severity and incidence. An analysis of a large prospectively case series (data collected by the US DILI Network) performed by Chalasani et al. [79] found that, in the first 300 cases, the group of cases with severe DILI had a greater proportion of diabetes (37%, versus 25% in the group of mild-to-moderate DILI). There were no between-group differences in BMI. The odds of severe DILI was 2.7 in the group with diabetes, independent of age, alcohol consumption, and whether the liver injury had a cytolytic or cholestatic pattern. El Serag and Everson [80] found that, after exclusion of patients with chronic liver disease, viral hepatitis, or congestive heart failure, veterans with type 2 diabetes have a greater risk (40%) of acute liver failure (including drug-induced). This finding suggests that whether it acts directly or indirectly, the presence of diabetes increases the risk of a severe injury when an acute liver injury (e.g., iatrogenic) occurs.

## 4. Relationships between Drugs for Metabolic Comorbidities and DILI

### 4.1. Hypolipidemics

Statins decrease the incidence and associated rates of mortality of peripheral vascular disease, stroke, and myocardial infarction [81]. Statins improve the lipid profile via the inhibition of hepatic 3-hydroxy-3-methylglutaryl coenzyme A (HMG-CoA) reductase. Lovastatin, simvastatin, pravastatin, atorvastatin, fluvastatin, and rosuvastatin are approved by the US Food and Drug Administration and are metabolized by CYP-450. Statins are the most often-prescribed class of lipid-lowering agents.

The approved statins are metabolized via hepatic clearance. Therefore, whether these drugs or their metabolites increase the risk of hepatic injury in individuals with underlying liver disease has been an issue of concern. The National Cholesterol Education Program (3^rd^ report) recommends that statin use is contraindicated in patients with active liver disease [82]. Statin manufacturers recommend that baseline serum liver enzymes are evaluated before a patient begins a statin and that statins should not be prescribed for patients with persistently elevated aminotransferase levels.

Statin use is associated with the development of idiosyncratic DILI. The Swedish Adverse Drug Reactions Advisory Committee found that statin-associated DILI affected 1.2/100,000 users; in most cases, a cholestatic/mixed pattern was present. Three patients experienced acute liver failure that resulted in death or liver transplantation [83, 84]. The Chalasani DILI study found that 2.6% of 899 cases of DILI could be attributed to statin use. There were subtle between-drug differences (atorvastatin 1.0%, rosuvastatin 0.8%, pravastatin 0.4%, lovastatin 0.2%, and fluvastatin 0.2%)[51]. The values for the incidence of mild-to-moderate and severe statin hepatotoxicity associated with atorvastatin, lovastatin, and simvastatin use were evaluated during a 6-month period in two large retrospective studies [85, 86].

A prospective study of 300 patients (Drug-Induced Liver Injury Network database) with suspected idiosyncratic DILI (regardless of causality scores) found that in only 14 (5%) patients, a statin alone or combined with another drug was implicated as the etiology of the DILI; there was no associated mortality [79]. Other results of clinical studies suggest that patients with NAFLD do not have a greater risk of statin-induced hepatotoxicity [87]. In one study, three groups of patients were compared. In first group (342 patients), they had hyperlipidemia and liver enzyme elevation and took statins. This group was compared with 1437 patients who had hyperlipidemia, but had normal aminotransferase levels; this group of patients also took statins and served as the statin control group. The third group consisted of 2245 patients who had elevated liver enzymes and were not taking statins. Compared with the liver disease controls who did not take statins, the first group did not have a higher incidence of mild-to-moderate (1.9% versus 4.7%, respectively, P = 0.2) or severe (0.4% versus 0.6%, respectively, P = 0.6) elevation in liver enzymes. The Pravastatin Pooling Project evaluated patients treated with pravastatin during a median period of 5 years [88]. Other pilot studies were used to evaluate atorvastatin, pravastatin, and rosuvastatin therapy over a period of variable duration (range, 6–21 months). The studies found that aminotransferase levels normalized in up to 78% of the patients [89–91]. Taken together, these results suggest that statin-associated hepatotoxicity is uncommon [84].

The results of experimental models suggest that statins have potentially beneficial liver effects. They increase nitric oxide production, targeting cholesterol at different points in the mevalonate pathway. They also protect from ischemia/reperfusion [92], hepatotoxicity [93], and drug-induced acute liver failure [94] by inhibiting drug-induced expression of genes involved in drug transport, the oxidative stress response, DNA repair, cell-cycle progression, and cell death.

An extremely rare event, statin-associated hepatotoxicity is generally associated with asymptomatic elevations in aminotransferase levels and results in mild clinical changes [95]. Study results indicate that statin use is safe and they should be used when indicated in patients with MetS or NAFLD, or both [51, 83, 85, 96]. They seem to provide protective effects in patients with NAFLD and nonalcoholic steatohepatitis who have liver injury [97, 98].

### 4.2. Antidiabetic Drugs

An important drug for T2DM treatment, metformin, is also used to treat patients with fatty liver [99]. Because it is not metabolized via the hepatic route, it is generally not considered to be toxic to the liver [100]; liver injury associated with the use of this drug is rare. Animal model studies found that metformin improves methotrexate-induced hepatotoxicity [101]. In the limited number of case studies, most patients with metformin-induced liver injury were also taking other potentially hepatotoxic drugs [102–104]. Only a few cases of metformin-induced liver injury without associated use of other hepatotoxic drugs have been reported [105, 106]. Idiosyncratic, direct, or drug-drug interaction [107] are possible mechanisms of injury that result in acute hepatocellular or cholestatic jaundice, or both.

T2DM is often treated using medications from the thiazolidinedione drug class. A higher incidence of hepatotoxicity and death led to the withdrawal of troglitazone, which was the first US Food and Drug Administration-approved thiazolidinedione [108–110]. Compared to troglitazone, the glitazones rosiglitazone and pioglitazone have similar efficacy for control of blood glucose levels in patients with T2DM. They are also considered to be less hepatotoxic. However, they were withdrawn from use in some countries due to apparent associated adverse cardiac effects [109, 110]. Impairment of mitochondrial function via inhibition of mitochondrial respiration, altered membrane potential, decreased ATP reserves, and increased oxidative stress are possible hepatotoxicity mechanisms found by in vitro studies [111–114]. Troglitazone has the highest potency to inhibit mitochondrial function [113].

The oral antidiabetic agents, acarbose [115] and gliclazide [116], have also been implicated in liver toxicity [108].

## 5. Conclusion

Drug-induced hepatotoxicity is a significant clinical problem. Patients with obesity, diabetes, or NAFLD have an increased risk of hepatotoxicity and of more severe hepatotoxicity. The associations between dyslipidemia and DILI require further examination. The complex interactions between deleterious and protective mechanisms of cytotoxicity in human hepatic steatosis are evident given the contrast between the many experimental results that indicate the presence of a heightened risk of hepatotoxicity in the fatty liver and the few recorded clinical cases of DILI in patients with NAFLD. Expansion of both clinical and basic research into the mechanisms of DILI is warranted. Special care is therefore needed to determine the associations between drugs used to treat metabolic disorders and DILI development.

## Figures and Tables

**Table 1:** Overview of host metabolism factors influencing specific mechanisms.

Mechanistic factors	Host metabolism factors	Host responses
Threshold dose	High body fat	Reduce drug clearance
Covalent binding	Alcohol, diet	Inducers/inhibitors of drug metabolizing enzymes
Oxidative stress	Obesity/insulin resistance/NAFLD, advanced cellular senescence	Increase cellular oxidants
	Fatty liver	Increase lipid peroxidation
	Obesity/insulin resistance/ NAFLD, nutrition	Depletion of antioxidants
Mitochondrial liability	Advanced cellular senescence (insulin resistance/NASH, chronic inflammation)	Mitochondrial dysfunction
Hepatic transporters inhibition	Altered hepatic FXR(e.g., NASH )	Hepatic transporter regulations
Inflammation and immune responses	Increased influx of LPS (e.g., alcohol abuse), obesity	Proinflammatory Conditions influence inflammation and immune response
Tissue injury and repair	Altered FXR, nutritional deficiencies	Influence tissue repair
